# Posttraumatic and Depressive Symptoms in Victims of Occupational Accidents

**DOI:** 10.1155/2012/184572

**Published:** 2012-05-29

**Authors:** Giulia Buodo, Caterina Novara, Marta Ghisi, Daniela Palomba

**Affiliations:** Department of General Psychology, University of Padova, Via Venezia 8, 35131 Padova, Italy

## Abstract

The present descriptive study was aimed at evaluating posttraumatic and depressive symptoms and their cooccurrence, in a sample of victims of workplace accidents. Also, posttraumatic negative cognitions were assessed. Eighty-five injured workers were evaluated, using the PTSD Symptom Scale, the Beck Depression Inventory II, and the posttraumatic Cognitions Inventory. 49.4% of injured workers reported both depressive and posttraumatic symptoms of clinical relevance. 20% only reported posttraumatic, but not depressive, symptoms, and 30.6% did not report either type of symptoms. The group with both posttraumatic and depressive symptoms displayed greater symptom severity and more negative cognitions about the self and about the world than the other two groups. The obtained findings indicate that workplace accidents can have a major impact upon the mental health of victims. Early interventions should be focused not only on the prevention or reduction of posttraumatic and depressive symptoms but also on restructuring specific maladaptive trauma-related cognitions.

## 1. Introduction

It has been consistently demonstrated that in some individuals exposure to traumatic events is followed by full or partial posttraumatic stress disorder (PTSD; see [1]). However, in a substantial proportion of cases PTSD is not the only psychological disorder occurring after a traumatic event. Indeed, survey studies report that lifetime comorbidity of PTSD with at least one other psychiatric disorder is found in about 70–80% of affected individuals [2, 3]. Among axis I disorders, major depression has the highest comorbidity rate with PTSD, with 30–50% of individuals affected by PTSD showing significant depressive symptoms [2, 4].

In order to explain the frequent cooccurrence of PTSD and depression, different hypotheses have been proposed. The possibility of a common underlying vulnerability to both disorders is suggested by the apparent bidirectional relationship between PTSD and depression, where preexisting depression can increase an individual's susceptibility to develop PTSD after a trauma and, on the other hand, PTSD can increase the onset probability of depression [2, 5–7]. The hypothesis of a shared diathesis is further strengthened by data showing an increased risk for major depression in individuals with PTSD, but not in trauma-exposed individuals without PTSD [1]. Therefore, these findings suggest that exposure to traumatic events in itself does not increase the risk for developing depression independent of PTSD effects.

Although the cooccurrence of PTSD and depression following trauma is the rule rather than the exception, it is worth noting that these two disorders can indeed occur independently of one another in a small but significant minority of cases (e.g., [8, 9]). This evidence runs against the idea that PTSD and depression are parts of a single general traumatic stress construct and rather suggests that they might be independent outcomes of traumatic events. Importantly in this respect, it has been reported that a two-factor model of PTSD and depression as correlated but independent conditions following trauma exposure provides the best fit of quantitative data measuring the two constructs [10] and that the combination of variables predicting depression is different from the group of factors predicting PTSD, at least in the acute aftermath of trauma [11]. Research on the types of attributional styles associated with differential psychological outcomes after traumatic events provides additional indications of disorder-specificity for PTSD and depression. The development of feelings of hopelessness and depression has been related to a helpless attributional style, in which negative events are attributed to global, stable, and internal causes (e.g., [12]). On the other hand, higher levels of PTSD symptoms after trauma exposure seem to be predicted by the tendency to attribute negative events to external, rather than internal, causes [12–15]. Besides attributional styles, maladaptive beliefs have also been suggested to play a symptom-specific role in the context of posttraumatic adjustment. Negative thoughts about the self (the beliefs about being weak and incompetent) have been found to uniquely correlate with PTSD severity even after controlling for the severity of concurrent depressive symptoms, whereas negative thoughts about the world (the belief that the world is entirely dangerous and people are untrustworthy) and self-blame (the perception of being responsible for the traumatic event) seem to share notable variance with depression in their association to the severity of posttraumatic symptoms [16]. These findings suggest that, beyond a number of non-specific cognitive factors, PTSD and depression might involve disorder-specific sets of cognitive distortions [17].

Lastly, subgroups of traumatized individuals who are diagnosed with PTSD only and comorbid PTSD and depression seem to differ with regard to subjective distress and social impairment. Individuals who meet diagnostic criteria for both PTSD and major depression have been found to report more severe posttraumatic, depressive, and anxiety symptoms, increased impulsivity, hostility and suicidality, and worse social functioning than those with PTSD alone (see [8, 10, 18]).

In view of the methodological heterogeneity and design limitations that inevitably exist in studies on PTSD-depression comorbidity, the issues of causality, temporal course and prognosis, commonality, or independence of the two disorders following trauma exposure remain a matter of debate.

Much of the existing research on PTSD, depression, and their comorbidity has considered such traumatized populations as combat veterans, victims of childhood sexual abuse, and survivals of natural disasters (e.g., [19–21]). More recently, the victims of occupational accidents are receiving increasing attention as a trauma group. Occupational accidents include a broad range of unforeseeable, sudden events occurring in the workplace, that cause the worker functional or physical disability of varying severity, whether temporary or permanent. Although accidents can occur in virtually every workplace, some occupations have a high and predictable risk of being exposed to threat, serious injuries or disasters, for instance, policemen, rescue personnel, firefighters, bank officers, or medical emergency personnel (e.g., [22, 23]). Therefore, most of the investigations on the psychological consequences of work-related injuries have considered such occupational categories. Other occupational settings, such as the industrial and the constructions sectors, where traumatic accidents occur frequently but that are not typically considered as high-risk settings, have received less systematic attention in this context. However, the relevant literature indicates that both victims of accidents in high-risk occupations and workers injured in industrial accidents show increased levels of emotional distress, anxiety and depression, subjective personal vulnerability, anger, irritability, somatic focus, preoccupations about the future, inactivity, and dependence [24–27]. Indeed, workplace accidents are increasingly reported as potentially traumatic events that may result in the development of acute stress disorder, adjustment disorder, or eventually PTSD [24, 25, 28–31]. A percentage of injured workers as high as 30–40% has been reported to show symptoms consistent with full or partial PTSD after the accident (see [29, 31]).

Research in this area is still rather limited. In particular, the comorbidity of posttraumatic and depressive symptoms in injured workers, although expected, remains underexplored. Although previous research indicates that comorbidity rates for PTSD and other disorders are similar across different trauma populations, the type of trauma appears to be an important variable affecting the type, extent, and course of PTSD comorbidity [32, 33]. In particular, the findings of studies on PTSD and depression comorbidity, mostly obtained in victims of intentional and/or repeated traumatic events such as domestic violence, sexual abuse and combat, may not warrant generalization to victims of single, nonintentional traumas such as workplace accidents. Indeed, intentional events are generally reported to be associated with worse posttrauma adjustment than non-intentional events [2], as events that are purposely inflicted are thought to be more difficult to cope with than are unintentional events [34].

Therefore, the aims of the present descriptive study were (a) to examine the proportion of victims of workplace accidents who report posttraumatic and/or depressive symptoms and (b) to assess whether subgroups of injured workers with posttraumatic and/or depressive symptoms would differ in symptom severity and in posttraumatic negative cognitions.

Based on the available literature on PTSD-depression comorbidity, and on the psychological sequelae of work-related accidents in particular, we hypothesized to show evidence of (a) clinically relevant symptoms of PTSD in a large proportion of injured workers, (b) the presence of comorbid depressive symptoms in a subgroup of injured workers with PTSD (c) greater symptom severity and more negative posttraumatic cognitions in individuals with comorbid PTSD and depressive symptoms than in individuals with PTSD or depression alone.

## 2. Methods

### 2.1. Participants

Eighty-five injured workers were recruited in several towns in Northern, Central, and Southern Italy among associates of the Associazione Nazionale Mutilati e Invalidi del Lavoro (ANMIL, a nonlucrative organization with several seats in Italy, that provides social, material, and moral support to individuals who sustained work accidents). ANMIL administrative staff members identified eligible participants by searching through the organization's database those who met the criteria established by the authors (see the following). The full database was used to avoid sampling bias. ANMIL staff members then contacted potential participants by telephone to preliminary assess their willingness to take part in the study and scheduled an appointment for those who agreed. ANMIL had no further involvement in data collection, analysis, and interpretation. The assessment procedure (see the following) was carried out by the authors and their collaborators.

Injured workers were eligible for participation if they met the following criteria: age between 18 and 60 years, time elapsed from the accident between 6 months and 10 years before the study, and degree of physical impairment between 25% and 75% (corresponding to a medium level of physical impairment), as evaluated by the Istituto Nazionale per l'Assicurazione contro gli Infortuni sul Lavoro (INAIL, Italian Workers' Compensation Authority).

Exclusion criteria were the following: use of drugs or medications that could influence the individual's ability to undergo the assessment procedure, presence of physical illnesses and psychopathologies unrelated to the accident, traumatic brain injury, and incapacity to give informed consent.

The sample included 78 males (91.8%) and 7 females (8.2%). Mean age was 38.3 years (S.D. = 7.7; range = 22–58) and mean years of education were 10.7 (SD = 2.7; range = 5–18). Marital status was as follows: 23 participants were single (27.1%), 43 were married (50.6%), 9 were cohabitant (10.6%), and 9 were divorced (10.6%).

Mean degree of impairment was 47.3% (S.D. = 15.5; range = 25–75%), and mean length of time since the accident was 5.3 years (S.D. = 2.3; range = 1–10). With regard to the occupational status after the accident, 55 participants (64.7%) were employed and 30 (35.3%) were unemployed.

### 2.2. Measures

All participants were administered the following measures.

1. A semistructured interview covering socio-demographic data (age, marital status, education, use of medication, presence of physical illnesses, etc.) and accident-related data (a description of the work accident, the degree of physical impairment, and the length of time since the accident). Also, the interview was aimed at ascertain the absence of other traumatic events beyond the workplace accident.
2. Beck Depression Inventory: second edition (BDI-II; [35]; Italian version by [29]) is a widely used self-administered questionnaire evaluating the severity of depressive symptoms during the last two weeks preceding the assessment. It consists of 21 items answered on a 0–3 scale. The Italian BDI-II cutoff score is 12 [36].
3. PTSD Symptom Scale (PSS; [37]) contains 17 items designed to measure the frequency of PTSD symptoms (according to DSM III-R). The respondents are asked to evaluate on a Likert scale (0: never; 3: five or more times per week) how often they have experienced each symptom in the past week. The items are grouped into three subscales: reexperiencing (detecting symptoms such as nightmares and flashbacks), avoidance (detecting symptoms such as detachment and loss of interests), and arousal (detecting symptoms such as irritability, difficulty concentrating, and hypervigilance). A total score is also derived, which reflects the severity of PTSD symptomatology (up to 10: mild; 11–20: moderate; 21–35: moderate to severe; above 36: severe; [38]).
4. Posttraumatic Cognitions Inventor (PTCI; [39]) is a 33-item self-report measure that assesses trauma-related thoughts and beliefs on a Likert scale (1: totally disagree; 7: totally agree). The PTCI is composed of three subscales: Negative Cognitions About Self, Negative Cognitions About the World, and Self-Blame.

The Italian translations of the PSS and the PTCI had been obtained from back translation by two expert English mother-tongue psychologists.

### 2.3. Procedure

All individuals participated on a voluntary basis. Before entering the study, they were informed of the study aims and gave their written consent. Each participant underwent the semistructured interview and was then requested to fill in the questionnaires. The order of administration of questionnaires was rotated across participants to control for order effects.

The study was conducted in compliance with the Declaration of Helsinki and approved by the institutional board of the participating institution.

### 2.4. Statistical Analyses

As a first step, injured workers were divided into groups, based on the scores obtained on the PSS and the BDI-II. Those who scored equal or above 11 on the PSS were classified as High PSS, and those who scored below 11 were classified as Low PSS. Individuals who scored equal or above 12 on the BDI-II were classified as High BDI, and those who scored below 12 as Low BDI.

Then, a multivariate analysis of variance (MANOVA) was performed to compare the groups on the following sociodemographic data: education, degree of impairment and time since the accident. Chi-squared analyses were conducted on gender, marital status, and employment status. Lastly, a multivariate analysis of covariance (MANCOVA) was performed to compare scores on self-report questionnaires between the three groups, using the time since the accident as a covariate.

When results were significant (*P* < .05), Student-Newman-Keuls (SNK) post hoc comparisons were applied to identify specific differences between groups.

## 3. Results

Overall, sixty-four percent (*N* = 59) of injured workers scored ≥11 on the PSS, corresponding to a moderate or more severe posttraumatic symptomatology, and 49.4% (*N* = 42) scored ≥12 on the BDI-II, corresponding to depressive symptoms of clinical relevance. Interestingly, all the individuals who scored above the cut-off on the BDI-II also had moderate or more severe posttraumatic symptoms (High PSS/High BDI, *N* = 42).

Another group of individuals scored ≥11 on the PSS and below the cut-off on the BDI-II (High PSS/Low BDI, *N* = 17).

A third group of individuals reported low scores on both questionnaires (Low PSS/Low BDI, *N* = 26).

There were no subjects scoring above the cut-off on the BDI-II and below 11 on the PSS.

These data are shown on Table 1.

As reported on Table 2, there were no differences between the three groups with regard to age, education, gender, marital status, and degree of physical impairment. The unemployment rate was lower in Low PSS/Low BDI participants as compared with the other two groups. The number of years since the accident was lower in the High PSS/High BDI group as compared with the High PSS/Low BDI and the Low PSS/Low BDI groups.

The analysis of clinical variables (see Table 3) showed that the High PSS/High BDI group obtained higher scores on all the three subscales of the PSS as compared with the other two groups, while the High PSS/Low BDI group scored higher than the Low PSS/Low BDI group only on the avoidance subscale.

The High PSS/High BDI group scored higher than the other two groups on the PTCI and on its subscales of Negative cognitions about self and Negative Cognitions About the World. The three groups did not differ on the Self-Blame subscale score.

Results did not change when entering the years since the accident as a covariate in the multivariate analysis (*F* = 0.78; *P* = ns). Therefore, Table 3 reports the results of the MANOVA.

## 4. Discussion

In the present descriptive study, we observed that a proportion as high as 64% of injured workers had moderate-to-severe posttraumatic symptoms. More in particular, three subgroups of victims of workplace accidents were identified on the basis of self-reported posttraumatic and depressive symptomatology: individuals with both posttraumatic and depressive symptoms (49.4%), individuals with PTSD symptoms only (20%), and trauma-exposed workers without either type of symptoms (30.6%). These three subgroups differed with respect to symptom severity and trauma-related cognitions, with the High PSS/High BDI group reporting greater severity of posttraumatic and depressive symptomatology, and more negative cognitions about the self and about the world than the High PSS/Low BDI and the Low PSS/Low BDI groups.

Several things emerge from our findings. Firstly, our data on the occurrence and severity of PTSD symptoms (with or without comorbid depression) converge with those of previous research on victims of occupational accidents (see [25]), and confirm that a sizeable proportion of injured workers experience significant posttraumatic stress symptoms. Secondly, our finding that depressive symptomatology cooccurred with posttraumatic symptoms in about half of participants indicates that comorbidity is as high among injured workers as it is in other trauma groups (see [8, 33]). The greater severity of posttraumatic symptoms in the High PSS/High BDI group than in the High PSS/Low BDI group is again in agreement with other studies in the trauma literature, reporting an association of PTSD-depression comorbidity with greater severity of posttraumatic symptoms [8, 10, 33]. Lastly, the finding that the group of injured workers with both PTSD and depressive symptoms experienced more negative thoughts about the self and about the world than the High PSS/Low BDI group fits with previous research reporting relatively more distorted trauma-related beliefs in individuals with both PTSD and depression as compared with individuals with PTSD only [40]. The absence of group differences with regard to the scorings on the Self-Blame subscale of the PTCI might have multiple reasons, that is, the poorer discriminant validity of this scale as compared with the other two scales (see [16]), or the possibility that in our sample of injured workers the development of psychopathology had no relationship with self-attribution of responsibility for the accident.

Overall, our findings largely converge with the existing literature on PTSD and depression following traumatic events, by showing that comorbidity is common also among victims of occupational accidents and that in these individuals comorbidity is accompanied by more severe PTSD symptoms and more maladaptive trauma-related cognitions. Although these results were somehow expected and may not be surprising, similarities across different trauma populations should not be taken for granted so much so that distinct trauma types have been found to be associated with different posttraumatic symptom presentations [41].

Although the present study indicates that workplace accidents are often followed by cooccurring PTSD and depression, it is worth pointing out that a subgroup of injured workers in our sample had PTSD symptoms without depression. This evidence supports the view that PTSD and depression might be separate outcomes of traumatic events, occurring independently of one another in a significant minority of cases (e.g., [8, 9]). In this regard, it has to be noted that the mean time elapsed from the accident was significantly longer in the High PSS/Low BDI group (6.31 years) than in the High PSS/High BDI group (4.63 years). This difference might be interpreted in terms of full or partial symptom remission, but a firm conclusion about this issue could only be drawn from longitudinal data. Importantly, however, the results of the present study did not change when the number of years since the accident was entered in the analyses as covariate, indicating that the observed group differences existed independent from the length of time elapsed from the accident. The High PSS/Low BDI group of injured workers endorsed more avoidance, but not reexperiencing and hyperarousal, symptoms as compared with the Low PSS/Low BDI group. Theoretical models of PTSD substantially agree in recognizing that avoidance of thoughts and feelings and/or external stimuli related to the trauma play a key role in the long-term maintenance of PTSD symptoms, since avoidance is thought to prevent adequate emotional processing of the traumatic event and to interfere with integration and restructuring of dysfunctional cognitions concerning the trauma (e.g., [42]). Our finding might indicate that in injured workers that have developed long-term PTSD symptoms without concurrent depressive symptoms, poor adjustment is specifically associated with persistent avoidance symptoms.

A substantial proportion of injured workers in our sample were relatively well adjusted following the workplace accident, as they did not show either posttraumatic or depressive symptoms of clinical relevance. Importantly, in the Low PSS/Low BDI group the mean degree of physical disability was comparable with that of the other two groups, whereas the rate of unemployment was significantly lower than that in the other two groups. These findings fit with recent observations that the objective severity of physical injury following a traumatic accident is not related to the occurrence or severity of the psychological consequences (e.g., [43]) and that the absence of psychopathological symptoms after a severe accidental injury is a positive prognostic factor for early return to work (e.g., [44, 45]). However, the design of this study does not provide a direct way of knowing the direction of causality, if any, between the absence of posttraumatic and depressive psychopathology and return to work. Further studies in the field of occupational health research should clarify which individual psychological factors best predict return to work following an injury, and develop tailored interventions for facilitating a timely return in the work force [46–49].

In contrast with other studies on PTSD, depression, and their comorbidity in different trauma populations, where subgroups of individuals with depression only were identified (e.g., [8, 11]), we did not observe a depression-only subgroup in our sample of injured workers. The reason for this may be in the length of time elapsed between the accident and the assessment. In the studies where depression was found as a separate disorder following the trauma, independent of PTSD, traumatized individuals were assessed between 1 and 3-4 months post-trauma. Differently, the participants in our sample had been injured one to ten years before the assessment. Indeed, the mean time from the accident was 4.63 years in the High PSS/High BDI group. Together, these findings suggest that, in the acute aftermath of trauma, depressive symptoms may develop independent of PTSD, whereas in the longer run a more general traumatic stress response, which includes both depressive and posttraumatic symptoms, seems to be more prevalent among trauma survivors. Of course, the intrinsic limitations of a descriptive study do not allow us to address crucial issues regarding the order of onset of cooccurring posttraumatic and depressive symptoms, or cause-effect relationships.

We recognize that some limitations need to be taken into account when interpreting the findings of the present study. Firstly, as a descriptive study it can only quantify, but it cannot explain, the causal relationships among the variables under consideration. Secondly, the majority of injured workers in our study were males, thus limiting the generalizability of findings to female victims of occupational accidents. Because gender differences in relation to PTSD have been reported (see [50]), further studies should consider this variable carefully. Lastly, we did not explicitly assess the workers' attributional styles and their assumptions regarding responsibility for their accidents. These important issues should be addressed in future research studies.

## 5. Conclusions

The results of the present descriptive study have the potential to improve our understanding of the cooccurrence of posttraumatic and depressive symptoms following workplace accidents. Overall, the obtained rates indicate that workplace accidents occurring in the industrial/constructions settings can have a major impact upon the mental health of victims. Early treatment interventions should be designed, focusing not only on the prevention or reduction of posttraumatic and depressive symptoms but also on restructuring specific maladaptive trauma-related cognitions.

## Figures and Tables

**Table 1:** Percentages of participants scoring high or low on the PSS (High = ≥ 11, Low = < 11) and on the BDI-II (High = ≥ 12, Low = < 12).

	High BDI	Low BDI
High PSS	49.4%	20.0%
Low PSS	0%	30.6%

**Table 2:** Demographic characteristics of the three groups (High PSS/High BDI, High PSS/Low BDI, Low PSS/Low BDI).

	High PSS/High BDI (*N* = 42)	High PSS/Low BDI (*N* = 17)	Low PSS/Low BDI (*N* = 26)	F or Chi (df)	*P*	Post-hoc SNK
Age; years (standard deviation)	38.92 (7.06)	36.19 (7.31)	38.22 (8.68)	0.73 (2,79)	ns	
Female gender; percent	3.5%	3.5%	1.2%	2.72 (2)	ns	
Education; years (standard deviation)	10.64 (2.85)	10.00 (2.44)	11.39 (2.74)	1.23 (2,79)	ns	
Marital status; percent married	28.2%	5.9%	16.5%	15.24 (6)	ns	
Occupation				6.14 (2)	0.03	
Employed	27.1%	11.8%	25.9%			
Unemployed	22.4%	8.2%	4.7%			
Degree of physical impairment; percent (standard deviation)	46.35% (15.99)	49.06% (17.52)	46.61% (12.70)	0.18 (2,79)	ns	
Time since the accident; years (standard deviation)	4.63 (2.28)	6.31 (1.99)	5.91 (2.08)	4.57 (2,79)	0.01	1 < 2,3*

*Note: 1 = High PSS/High BDI; 2 = High PSS/Low BDI; 3 = Low PSS/Low BDI.

**Table 3:** Clinical characteristics of the High PSS/High BDI, High PSS/Low BDI, and Low PSS/Low BDI groups. Mean scores (standard deviations) are reported for each questionnaire.

	High PSS/High BDI (*N* = 42)	High PSS/Low BDI (*N* = 17)	Low PSS/Low BDI (*N* = 26)	F or Chi (df)	*P*	Post-hoc SNK
BDI-II	22.38 (7.33)	7.31 (3.45)	4.50 (3.19)	92.16 (2,79)	0.001	1 > 2,3*
PSS Total	24.90 (8.52)	18.31 (6.94)	3.65 (2.92)	75.39 (2,79)	0.001	1 > 2 > 3
Re-experiencing	8.33 (6.57)	6.44 (4.17)	0.74 (1.09)	16.37 (2,79)	0.001	1 > 2,3
Avoidance	9.95 (3.93)	6.81 (3.27)	1.43 (1.37)	49.92 (2,79)	0.001	1 > 2> 3
Hyperarousal	7.80 (3.71)	5.94 (3.39)	1.57 (1.83)	27.58 (2,79)	0.001	1 > 2,3
PTCI Total	118.30 (35.03)	84.63 (30.49)	72.74 (20.40)	18.21 (2,79)	0.001	1 > 2,3
Negative Cognitions About the Self	72.93 (23.77)	48.94 (19.23)	38.43 (14.41)	22.47 (2,79)	0.001	1 > 2,3
Negative Cognitions About the World	31.83 (9.13)	24.56 (11.41)	24.57 (8.51)	5.81 (2,79)	0.001	1 > 2,3
Self-Blame	13.55 (7.38)	11.12 (5.31)	9.74 (6.24)	3.04 (2,79)	ns	

*Note: 1 = High PSS/High BDI; 2 = High PSS/Low BDI; 3 = Low PSS/Low BDI.
